# Hoarding symptoms among psychiatric outpatients: confirmatory factor analysis and psychometric properties of the Saving Inventory – Revised (SI-R)

**DOI:** 10.1186/s12888-016-1043-y

**Published:** 2016-10-26

**Authors:** Siau Pheng Lee, Clarissa Ong, Vathsala Sagayadevan, Rebecca Ong, Edimansyah Abdin, Susan Lim, Janhavi Vaingankar, Louisa Picco, Swapna Verma, Siow Ann Chong, Mythily Subramaniam

**Affiliations:** 1 Institute of Mental Health, Singapore, Singapore; 2 The Chinese University of Hong Kong, Shatin, Hong Kong SAR, NT People’s Republic of China; 3 Present address: Department of Psychology, Utah State University, 2810 Old Main Hill, Logan, UT 84322-2810 USA; 4 Present address: Institute of Mental Health, 10 Buangkok View, Singapore, 539747 Singapore; 5 Research Division, Institute of Mental Health, 10 Buangkok View, Singapore, 539747 Singapore

**Keywords:** Psychometric properties, Validity, Cultural influence in psychiatry, Asian, Singapore, Difficulty discarding, Clutter, Excessive acquisition

## Abstract

**Background:**

The growing interest in problematic hoarding as an independent clinical condition has led to the development of the Saving Inventory-Revised (SI-R) to assess hoarding phenomenology. The SI-R is one of the most widely used instruments to measure hoarding symptoms; however, it lacks validation in non-Western samples.

**Methods:**

The current study examined the construct, convergent, and discriminant validity of the SI-R among 500 outpatients at a psychiatric hospital in Singapore. The three-factor structure solution of the SI-R was fitted in a confirmatory factor analysis.

**Results:**

The final model achieved mediocre fit (*χ*2 = 1026.02, df = 186; RMSEA = 0.095, SRMR = 0.06; CFI = 0.86; NNFI = 0.85). Two reverse-coded items (items 2 and 4) were removed due to insufficient factor loadings, resulting in the modified 21-item SI-R (SIR-21). Our findings indicate the need to further examine the construct validity of the SI-R, particularly in non-Western samples. Nonetheless, correlations with other hoarding-related constructs, such as anxiety (Beck Anxiety Inventory) and depression (Beck Depression Inventory-II), supported the convergent and discriminant validity of the SIR-21 in our sample.

**Conclusions:**

Findings in our current majority Chinese sample were consistent with previous observations from other Chinese samples. Implications were discussed from a cross-cultural perspective, such as cultural emphasis on saving for future use and overlap between the concepts of discarding and acquiring in Chinese samples. Future studies should also examine differences among other ethnic groups (e.g., Malay, Indian).

## Background

Hoarding disorder is characterized by (1) persistent difficulty discarding possessions, regardless of the true value of possessions, (2) resulting clutter that prohibits intended use of living spaces, (3) strong urges to save items or distress in response to discarding objects, (4) distress to self and/or others resulting from failure to maintain a safe environment due to clutter (*Diagnostic and Statistical Manual of Mental Disorders, Fifth Edition* [DSM-5]) [1]. Excessive acquisition – which includes excessive buying, acquisition of free items, and, more rarely, stealing – presents frequently among individuals with hoarding symptoms; it is stipulated as a specifier, rather than a diagnostic criterion in the DSM-5 [1–4].

Epidemiological studies revealed at least 2–5 % of the general population present with problematic hoarding [5, 6]. Significant impairments brought about by hoarding behavior, such as relationship tension with those sharing the same living space [7], impairment in quality of life, and daily functioning [8], health, safety and hygiene concerns resulting from clutter [9], suggest that this phenomenon needs attention at both a patient management and policy level.

The growing interest in hoarding disorder has brought about the development of several instruments to measure its symptomatology. The Structured Interview for Hoarding Disorder [10] was designed to evaluate the presence of DSM-5 hoarding disorder, whereas other scales, such as the Hoarding Rating Scale (HRS) [11] and Saving Inventory-Revised (SI-R) [12] have been used to evaluate significant hoarding [13, 14]. In particular, the SI-R is one of the most widely used measures of hoarding severity due to its strong psychometric properties and theoretically guided development [12, 15]. That is, items on the SI-R were first created to ensure accurate representation of the phenomenology of hoarding, and then refined based on results from factor analyses [12]. The original validation study of the SI-R indicated 3 factors that were consistent with the 3 domains of hoarding: *Difficulty Discarding*, *Clutter*, and *Acquisition*.

Yet, validation of the SI-R has been largely done in Western samples (e.g. [16–19]). There is growing evidence that cultural differences exist in comprehending, interpreting, and responding to the language written in describing psychiatric symptoms [20]. Manifestation of psychiatric symptoms may also vary across cultures [20]. Given that risk factors associated with psychiatric disorders may not be generalizable across cultures [21], it is important to examine the appropriateness of the SI-R in capturing hoarding behaviors across different cultures.

Although the construct validity of SI-R has been investigated in several previous studies [16–19], a recent study conducted in a Chinese college sample by Timpano et al. [22] did not replicate the 3-factor construct using the Mandarin version of SI-R, suggesting that the factor structure of the SI-R may be different in the Chinese population. Furthermore, the 2 items on self-control in the original SI-R were removed in the Chinese version due to low factor loading. The poor model fit could indicate differences in the (perceived or actual) role of self-control in hoarding symptomatology between European and Chinese samples. The specific nature of their sample (i.e., Chinese students) and lack of qualitative measures preclude any definite conclusions about cultural differences in the presentation of hoarding symptoms. Similar to Timpano et al.’s [22] results, Tang et al. [23] did not manage to get a good model fit when fitting the 3-factor structure of Frost et al.’s [12] original model in their confirmatory factor analysis in a Chinese college sample.

Given the limited research on hoarding in Asia, and preliminary evidence on cultural variance in its factor structure, further research is needed to understand how hoarding symptoms manifest in different cultural contexts. In the current study, we aimed to evaluate the factor structure, internal reliability, convergent validity, and divergent validity of the SI-R among psychiatric outpatients in Singapore. Singapore is an island nation in the southern part of Southeast Asia, and is often considered one of the cleanest cities in the world. The majority of its residents are first to fourth generation immigrants from other regions of Asia (i.e. China, India, etc.), and indigenous Malays [24]. Even after the British colonial period, cultural values from these origins are still strongly embedded in the daily life of the current generation [24]. In addition, the majority of Singapore residents stay in public housing developed by the Housing Development Board (HDB), a statutory board of the Singapore government [25]. Family members usually stay in the same household, and different households are within close proximity under this housing arrangement. Public housing is managed by the HDB to ensure its livability. Routine management such as cleaning of public areas and maintenance of common facilities are done by the town council. This housing arrangement makes the current sample a unique setting for the presentation of hoarding behavior. Additionally, according to the DSM-5, hoarding disorder is considered a distinct disorder and no longer a subtype of OCD [1], as hoarding behavior does not only present within the context of OCD [26–28]. Yet, previous studies on hoarding symptoms have largely focused on patients with OCD (see [12, 29]). Little is known about hoarding symptoms among psychiatric patients with other diagnoses. Hence, in the current study, we aimed to evaluate the factor structure, internal reliability, convergent validity, and divergent validity of the SI-R using a Singaporean psychiatric sample.

## Method

### Sample

Five hundred psychiatric outpatients were recruited from the Institute of Mental Health (IMH), a tertiary psychiatric hospital in Singapore, and its satellite clinics. To be eligible for the study, participants had to: 1) be at least 21 years old; 2) receive a primary DSM-IV diagnosis of any anxiety disorder, any depressive disorder, schizophrenia or pathological gambling from a clinician; 3) be able to understand and complete the study questionnaires in English; and 4) be cognitively capable of providing informed consent. Patients with intellectual disability and/or other forms of cognitive deficits were not recruited. None of the participants had formally received a diagnosis of hoarding disorder from a clinician.

Ethics approval was obtained from the institutional review board (Domain Specific Review Board, National Healthcare Group) prior to study commencement. Participants provided informed consent in written format before proceeding to complete the questionnaire. Participants were compensated 30 Singapore dollars on completion of the questionnaire.

### Instruments

The Saving Inventory-Revised (SI-R) [12] is a 23-item self-report questionnaire measuring hoarding-related experiences during the past week. Items include: ‘How much of your home does clutter prevent you from using?’ and ‘How distressing do you find the task of throwing things away?” Items are scored on a 5-point Likert scale (0 to 4), with higher scores indicating greater hoarding severity.

The Clutter Image Rating (CIR) scale [30] is a pictorial rating scale, consisting of 3 sets of 9 color images. Each set of images depicts a room (living room, bedroom, kitchen) with different levels of clutter (1 = least cluttered, 9 = most cluttered). Participants are required to select the image that most accurately reflects the amount of clutter in their homes.

The Saving Cognitions Inventory (SCI) [31] is a 24-item self-report questionnaire measuring maladaptive beliefs and emotional attachment towards possessions. The scale measures 4 facets: emotional attachment, control, responsibility, and memory. Participants were requested to indicate the extent to which they identified with each statement when deciding to discard something during the past week. Items are scored from 1 (not at all) to 7 (very much). Items include: ‘I could not tolerate if I were to get rid of this.’ and ‘Losing this possession is like losing a friend.’

The Activities of Daily Living Scale for Hoarding (ADL-H) [32] is a 15-item self-report questionnaire evaluating the extent to which clutter in the home prevents one from carrying out daily activities. Participants were requested to indicate the degree of difficulty they experienced performing the activities listed due to clutter or their hoarding problem. Items are scored on a 5-point Likert scale from 1 (can do it easily) to 5 (unable to do) and included activities such as ‘prepare food,’ ‘move around inside the house,’ and ‘sleep in bed.’

The Beck Anxiety Inventory (BAI) [33] is a 21-item inventory measuring distress associated with common symptoms of anxiety. Items are scored from 0 (not bothered at all) to 3 (severely bothered), with higher ratings indicating greater anxiety severity.

The Beck Depression Inventory-II (BDI-II) [34] is a 21-item inventory measuring depressive symptoms. Items are scored from 0 to 3, with higher ratings indicating greater depression severity.

### Statistical analyses

Descriptive statistics were calculated using SPSS v20.0, and a confirmatory factor analysis (CFA) was performed using R studio Mac version 0.99.484, ‘sem’ package version 3.1-6 [35].

### Confirmatory factor analysis

**Fig. 1:**
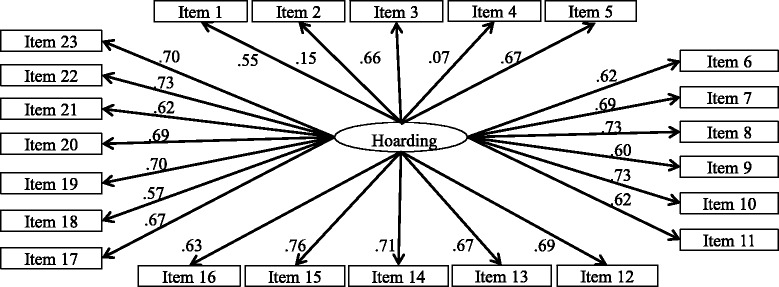
Unidimensional model – all 23 items loaded on a single factor. Refer to Table 2 for factor loadings. Error terms were omitted for simplicity of presenting

**Fig. 2:**
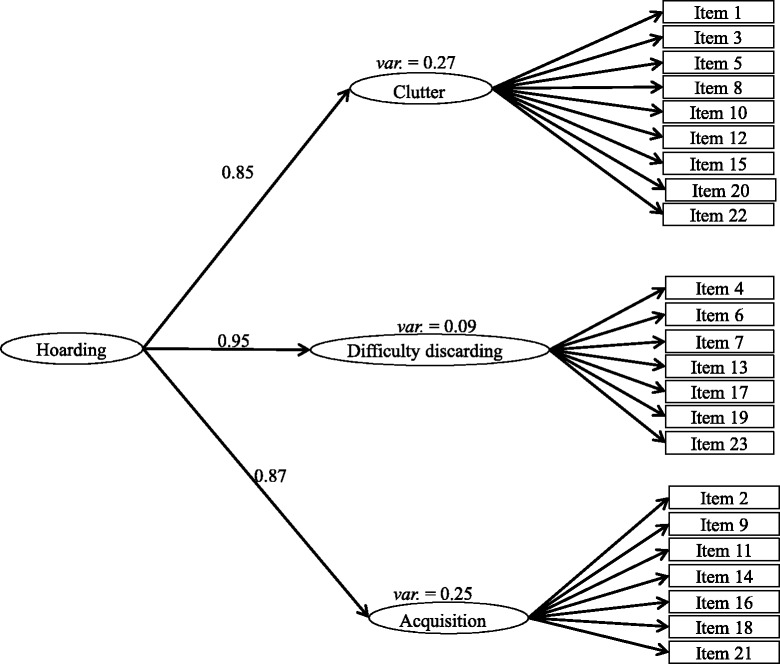
Second-order model – 23 items loaded on respective factors proposed by Frost et al. (2004), and three factors (i.e. Difficult discarding, Clutter, Acquisition) loaded on a single latent Hoarding factor. Error terms were omitted for simplicity of presenting

**Fig. 3:**
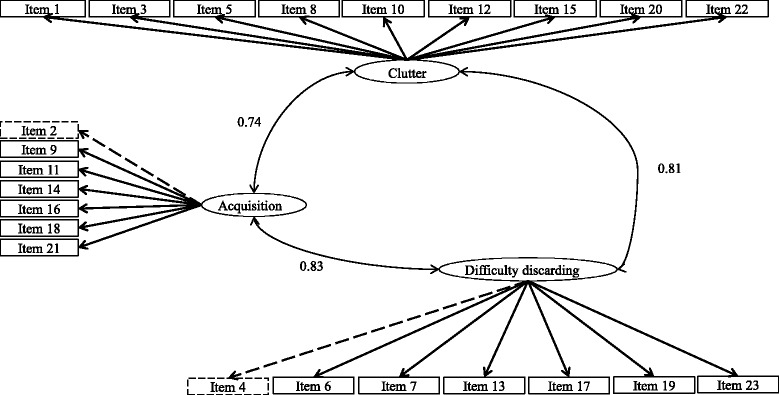
First-order model – items loaded on their respective latent factors as in Frost et al. (2004)’s model, and all three latent factors (i.e. Difficult discarding, Clutter, Acquisition) correlated with each other. Path coefficients as shown were obtained from 21-item solution. Item 2 and Item 4 were removed in the final CFA solution, hence are presented with dashed lines. Error terms were omitted for simplicity of presenting

Three models were fitted using CFA, they were (1) a unidimensional model – all 23 items loaded on a single factor (see Fig. 1); (2) a second-order model – 23 items loaded on respective factors proposed by Frost et al. [12], and three factors (i.e. difficult discarding, clutter, acquisition) loaded on a single latent Hoarding factor (see Fig. 2); (3) a first-order model – items loaded on their respective latent factors as in Frost et al.’s [12] model, with all three latent factors correlated with each other (see Fig. 3).

Maximum likelihood estimation method was used in CFA. Indices to evaluate model fit included non-normed fit index (NNFI), also known as the Tucker-Lewis Index (TLI) [36], comparative fit index (CFI) [37], standardized root mean square residual (SRMR), and root mean square error of approximation (RMSEA) [38]. Model fit was not evaluated solely based on chi-square, given that the chi-square index is greatly affected by sample size (i.e., tendency to over reject the model when sample size is large [39]). However, chi-square provides a descriptive measure for the overall model fit, hence it is reported in this paper. According to Hu and Bentler’s [40] recommendation, a model with NNFI ≥ 0.95, CFI ≥ 0.95, SRMR ≤0.08 and RMSEA ≤ 0.06 is considered a good fit, whereas MacCallum et al. [41] suggested that RMSEA values ranging between 0.08 and 0.10 indicate mediocre fit. Modification indices were also obtained to determine the additional relationship of model improvement beyond model specification. In addition, following Hair et al.’s [42] recommendation, for sample sizes of 500, a factor loading of at least 0.3 is considered significant. Thus, items with factor loadings lower than 0.3 were removed.

### Reliability and validity

SI-R subscale scores were calculated by averaging all the items retained in the confirmatory factor analysis. Item-specific multiple correlations, item-total correlations, and Cronbach’s alpha if item deleted were also obtained for each subscale.

Pearson correlations between SI-R subscales and the SCI, CIR, ADL-H, BAI, BDI-II were obtained to establish convergent and discriminant validity. Strong positive correlations between hoarding-related constructs (i.e., SCI, CIR, and ADL-H) and the revised SI-R indicate convergent validity, whereas low correlations between the BAI and BDI-II and the revised SI-R suggest discriminant validity.

## Results

### Participants

The sociodemographic distribution of participants is described in Table 1. The majority of participants were of Chinese ethnicity, had received at least secondary level education, were currently employed (including full-time and part-time employment), and staying in a HDB flat with their immediate family.

**Table 1:** Sociodemographic distribution of participants

	Number	Percent
Age		
Mean	35.29	
SD	10.1	
Gender		
Male	282	56.4
Female	218	43.6
Ethnicity group		
Chinese	351	70.2
Malay	50	10
Indian	67	13.4
Others	32	6.4
Diagnosis		
Anxiety disorders	144	28.8
Depressive disorders	153	30.6
Schizophrenia	150	30
Pathological gambling	53	10.6
Education level		
No formal/primary	23	4.6
Secondary/O-level	147	29.4
A-level	40	8
Polytechnic/diploma	196	39.2
University	94	18.8
Marital status		
Never married	309	61.9
Currently married	132	26.5
Divorced/separated	51	10.2
Widowed	7	1.4
Employment status		
Employed	281	56.8
Economically inactive	83	16.8
Unemployed	131	26.5
Living arrangement		
Staying alone	43	8.7
Roommate(s)	37	7.4
Spouse/non-married partner	43	8.7
Extended family	26	5.2
Immediate family	344	69.2
Resident type		
Bungalow/terrace	15	3
Private condo/flat	35	7.1
HDB^a^	433	87.7
Nursing home^b^	11	2.2

^a^Housing and Development Board (HDB) flats are a type of public housing managed by the HDB, a statutory board of the Singapore government. The majority of Singapore residents stay in HDB flats, which range in size from 1 to 5 rooms

^b^Nursing homes are a type of housing where stay-in-care is provided to psychiatric patients with milder clinical symptoms

Participants were categorized into one of four diagnostic groups based on their primary diagnosis — anxiety disorders, depressive disorders, schizophrenia, and pathological gambling. Within anxiety disorders, 49 (9.8 %) had obsessive compulsive disorder, 25 (5.0 %) had panic disorder, 20 (4.0 %) had generalized anxiety disorder, 11 (2.2 %) had social phobia, 1 (0.2 %) had specific phobia, and 38 (7.6 %) had anxiety disorder not otherwise specified (NOS). Within the depressive disorder group, 124 (24.8 %) had major depressive disorder, 17 (3.4 %) had dysthymia, and 12 (2.4 %) had depressive disorder NOS. Among the participants, 33 (6.6 %) had a comorbid anxiety disorder (specifically from each diagnostic group: anxiety disorders, *n* = 15; depressive disorders, *n* = 13; schizophrenia, *n* = 4; pathological gambling, *n* = 1), 47 (9.4 %) had a comorbid depressive disorder (specifically from each diagnostic group: anxiety disorders, *n* = 32; schizophrenia, *n* = 6; pathological gambling, *n* = 9), and 9 (1.8 %) had comorbid anxiety and depressive disorders (specifically from each diagnostic group: anxiety disorders, *n* = 7; schizophrenia, *n* = 1; pathological gambling, *n* = 1).

### Confirmatory factor analysis

The unidimensional model (see Fig. 1) did not achieve a satisfactory fit (chi square = 1726.50, df = 230, *p* < .05; RMSEA = 0.11, SRMR = 0.08, NNFI = 0.74, CFI = 0.76), indicating that the SI-R was not represented by a single latent factor in the current sample. Hence, we proceeded to the second-order model (see Fig. 2), which was empirically under-identified despite a positive number of degrees of freedom (chi-square = 1226.5, df = 224, *p* < .05; RMSEA = 0.095, SRMR = 0.07, NNFI = 0.82, CFI = 0.84), suggesting the phenomenon of *Empirical Under-identification* in CFA [43]. Refer to Table 2 for the factor loadings.

**Table 2:** Confirmatory factor analysis of Saving Inventory-Revised (23 items)

			Model 1: Unidimensional model	Model 2: Second order model	Model 3: First order model	
Model specification			23 items	23 items	23 items	21 items
	Mean	SD	Factor loading	Factor loading	Factor loading	Factor loading
Clutter						
item 1: How much of the living area in your home is cluttered with possessions?	1.74	1.03	0.55	0.60	0.60	0.60
item 3: How much of your home does clutter prevent you from using?	1.23	1.04	0.66	0.75	0.75	0.75
Item 5: How much of your home is difficult to walk through because of clutter?	0.96	1.03	0.67	0.75	0.75	0.75
Item 8: To what extent do you have so many things that your room(s) are cluttered?	1.32	1.06	0.73	0.75	0.75	0.75
Item 10: How much does clutter in your home interfere with your daily functioning?	1.10	1.11	0.73	0.78	0.78	0.78
Item 12: To what extent does clutter in your home cause you distress?	1.22	1.14	0.69	0.69	0.69	0.69
Item 15: To what extent do you feel unable to control the clutter in your home?	1.19	1.15	0.76	0.77	0.77	0.77
Item 20: How frequently does clutter in your home prevent you from inviting people to visit?	1.17	1.26	0.69	0.73	0.73	0.73
Item 22: To what extent does the clutter in your home prevent you from using parts of your home for their intended purpose?	0.96	1.05	0.73	0.75	0.75	0.75
Difficulty discarding						
^a^Item 4 (r): How much control do you have over your urges to save possessions?	1.77	1.12	0.07	0.05	0.05	
Item 6: To what extent do you have difficulty throwing things away?	1.32	1.14	0.62	0.69	0.69	0.69
Item 7: How distressing do you find the task of throwing things away?	1.27	1.15	0.69	0.75	0.75	0.75
Item 13: How strong is your urge to save something you know you may never use?	1.31	1.08	0.67	0.71	0.71	0.71
Item 17: How often do you avoid trying to discard possessions because it is too stressful or time consuming?	1.51	1.14	0.67	0.73	0.73	0.73
Item 19: How often do you decide to keep things you do not need and have little space for?	1.49	1.05	0.7	0.75	0.75	0.75
Item 23: How often are you unable to discard a possession you would like to get rid of?	1.30	1.13	0.7	0.72	0.72	0.72
Acquisition						
^a^Item 2 (r): How much control do you have over your urges to acquire possessions?	1.68	1.09	0.15	0.18	0.18	
Item 9: How distressed or uncomfortable would you feel if you could not acquire something you wanted?	1.66	1.16	0.60	0.67	0.67	0.67
Item 11: How strong is your urge to buy or acquire free things for which you have no immediate use?	1.29	1.15	0.62	0.72	0.72	0.72
Item 14: How upset or distressed do you feel about your acquiring habits?	1.20	1.13	0.71	0.73	0.73	0.72
Item 16: To what extent has your saving or compulsive buying resulted in financial difficulties for you?	1.28	1.21	0.63	0.71	0.71	0.71
Item 18: How often do you feel compelled to acquire something you see?	1.54	1.05	0.57	0.72	0.72	0.72
Item 21: How often do you actually buy (or acquire for free) things for which you have no immediate use or need?	1.36	1.04	0.62	0.71	0.71	0.71
Model fit						
Chi-square			1726.5	1226.5	1226.5	1026.02
^b^df			230	224	227	186
RMSEA			0.11	0.095	0.094	0.095
SRMR			0.08	0.07	0.07	0.06
NNFI			0.74	0.82	0.82	0.85
CFI			0.76	0.84	0.84	0.86

^a^Item 2 and Item 4 are reverse coding items

^b^df = degree of freedom, RMSEA = root mean square error of approximation, SRMR = standardized root mean square residual, NNFI = non-normed fit index, CFI = comparative fit index

Subsequently, we tested the first-order model (see Fig. 3) with the 23 SI-R items. The CFA on the 23-item SI-R achieved poor model fit based on a few indices (chi square = 1226.5, df = 227, *p* < .05; RMSEA = 0.094, NNFI = 0.82, CFI = 0.84), though SRMR of the model suggested acceptable fit (SRMR = 0.07). A closer look at the standard estimates (factor loadings) of each variable loading on the latent factor proposed by Frost et al. [12] revealed that all items except for item 2 (factor loading = 0.14) and item 4 (factor loading = 0.03) had factor loadings of at least 0.6 (see Table 2 for details). In line with Hair et al.’s [42] suggestion of minimum required factor loading, we removed these two items with low factor loadings from subsequent analyses.

A follow-up CFA on the 21-item SI-R did not achieve satisfactory model fit, although marginal improvement was observed (chi square = 1026.02, df = 186, *p* < .05; CFI = 0.86, SRMR = 0.06). In fact, RMSEA increased by a negligible extent, when compared with model 1 (RMSEA = 0.0947; in the previous model, RMSEA = 0.0945). Four pairs of items were suggested by modification indices to covary (item 3 and item 5, item 6 and item 7, item 12 and item 15, item 20 and item 22) in this solution. However, we did not further covary these four pairs of items due to lack of theoretical justification.

Subsequently, we formed subscales based on the CFA solution of the remaining 21 items (SIR-21). Three subscales consistent with Frost et al.’s [12] factor structure were formed: Clutter subscale (SIR-21-C), Difficulty discarding subscale (SIR-21-D), and Acquisition subscale (SIR-21-A).

### Reliability and validity

Cronbach’s alpha of all SIR-21 subscales ranged from 0.85 to 0.94, indicating good to excellent internal reliability (see Table 3).

**Table 3:** Reliability of items retained in final confirmatory factor analysis and exploratory factor analysis

Factor	within each subscale			SIR-21		
	Item-total correlation	Squared multiple correlation	Cronbach's α if item deleted	Item-total correlation	Squared multiple correlation	Cronbach's α if item deleted
SIR-21-C (Clutter)						
Item 1	0.58	0.40	0.90	0.52	0.42	0.94
Item 3	0.72	0.57	0.89	0.61	0.58	0.94
Item 5	0.70	0.56	0.89	0.60	0.57	0.94
Item 8	0.69	0.51	0.89	0.70	0.58	0.94
Item 10	0.72	0.55	0.89	0.69	0.58	0.94
Item 12	0.62	0.49	0.90	0.65	0.53	0.94
Item 15	0.72	0.57	0.89	0.72	0.62	0.94
Item 20	0.67	0.50	0.89	0.64	0.55	0.94
Item 22	0.68	0.51	0.89	0.68	0.56	0.94
SIR-21-D (Difficulty discarding)						
Item 6	0.65	0.54	0.84	0.59	0.57	0.94
Item 7	0.71	0.57	0.83	0.67	0.62	0.94
Item 13	0.63	0.42	0.85	0.65	0.52	0.94
Item 17	0.66	0.47	0.84	0.65	0.53	0.94
Item 19	0.67	0.49	0.84	0.69	0.58	0.94
Item 23	0.62	0.42	0.85	0.66	0.51	0.94
SIR-21-A (Acquisition)						
Item 9	0.59	0.37	0.84	0.58	0.44	0.94
Item 11	0.66	0.45	0.83	0.61	0.52	0.94
Item 14	0.62	0.43	0.83	0.69	0.58	0.94
Item 16	0.66	0.46	0.83	0.61	0.50	0.94
Item 18	0.68	0.51	0.82	0.57	0.56	0.94
Item 21	0.65	0.48	0.83	0.60	0.56	0.94

*SIR-21* 21-item Saving Inventory-Revised, *SIR-21-C* 21-item Saving Inventory-Revised Clutter subscale, *SIR-21-D* 21-item Saving Inventory-Revised Difficulty Discarding subscale, *SIR-21-A* 21-item Saving Inventory-Revised Acquisition subscale

Table 4 shows bivariate Pearson correlations between the SIR-21 subscales and related constructs. With regard to discriminant validity of the SIR-21, the SIR-21 subscales showed stronger relationships with hoarding-related constructs (i.e. SCI, CIR, ADL-H) than with the BAI and BDI-II. This provides evidence for convergent validity of the SIR-21 subscales in measuring hoarding symptoms. However, the relationship between the SIR-21 subscales (SIR-21-D and SIR-21-A) and ADL-H were comparable in strength (Pearson’s rho range; 0.28 to 0.31) to those between the SIR-21 subscales and the BAI and BDI-II (Pearson’s rho range; 0.26 to 0.34), with the exception of the SIR-21-C which showed a higher correlation (Pearson’s rho = 0.42) with the ADL-H than the BAI (Pearson’s rho = 0.23) and BDI-II (Pearson’s rho = 0.22).

**Table 4:** Correlations between 21-item Saving Inventory-Revised (SIR-21), 17-item Saving Inventory-Revised (SIR-17) and related constructs

	Mean	SD	Cronbach’s alpha	Correlation			
				SIR-21-C	SIR-21-D	SIR-21-A	SIR-21
SIR-21-C	1.21	0.82	0.90	1			
SIR-21-D	1.37	0.86	0.86	.71*	1		
SIR-21-A	1.39	0.86	0.85	.63*	.71*	1	
SIR-21	1.31	0.75	0.94	.91*	.89*	.86*	1
SCI-emotional attachment	27.03	14.44	0.93	.48*	.61*	.56*	.61*
SCI-control	11.71	5.25	0.78	.32*	.41*	.42*	.42*
SCI-responsibility	17.54	8.31	0.84	.50*	.60*	.60*	.63*
SCI-memory	13.46	6.92	0.81	.52*	.59*	.55*	.62*
SCI-total	69.17	31.61	0.96	.51*	.63*	.60*	.64*
CIR-kitchen	1.70	0.93	NA^a^	.45*	.38*	.31*	.44*
CIR-bedroom	1.67	1.01	NA^a^	.45*	.41*	.33*	.45*
CIR-living room	1.60	1.02	NA^a^	.49*	.38*	.36*	.47*
CIR-composite	1.65	0.85	0.84	.54*	.46*	.40*	.53*
ADL-H	1.40	0.63	0.96	.42*	.31*	.28*	.39*
BAI	16.94	13.35	0.95	.23*	.26*	.31*	.29*
BDI-II	19.64	13.77	0.94	.22*	.31*	.34*	.32*

*SIR-21* 21-item Saving Inventory-Revised, *SIR-21-C* 21-item Saving Inventory-Revised Clutter subscale, *SIR-21-D* 21-item Saving Inventory-Revised Difficulty Discarding subscale, *SIR-21-A* 21-item Saving Inventory-Revised Acquisition subscale, *SCI* Saving Cognition Inventory, *CIR* Clutter Image Rating, *ADL-H* Activities of Daily Living Scale for Hoarding, *BAI* Beck Anxiety Inventory, *BDI-II* Beck Depression Inventory – II

^a^NA = not applicable, due to only single item

*significant at *p* < .01 level

With respect to convergent validity with other hoarding-related constructs, the SIR-21-C showed a stronger relationship with the CIR than with the SIR-21-D and SIR-21-A. However, the opposite pattern was found with the SCI. That is, the SIR-21-D and SIR-21-A showed stronger relationships with SCI, than the SIR-21-C. These findings suggested that the SIR-21-C measures clutter-related consequences. The findings also indicated that the SIR-21-D and SIR-21-A measure hoarding-related cognitions, emotional attachment, and behavior.

## Discussion

The current study examined the psychometric properties of the SI-R among psychiatric outpatients with four major psychiatric diagnoses (anxiety disorders, depressive disorders, schizophrenia, and pathological gambling), at a tertiary psychiatric hospital in Singapore. It is the first to examine psychometric properties of the SI-R among outpatients with diverse psychiatric diagnoses in a Southeast Asian population. Construct validity, convergent validity, discriminant validity, and internal reliability were examined using CFA. The SIR-21 and its subscales (SIR-21-A, SIR-21-D, SIR-21-C) showed discriminant validity with the BAI and BDI-II, and convergent validity with other hoarding constructs (i.e., CIR, SCI, ADL-H). The SIR-21 and its subscales also showed good internal reliability. These results show that the SIR-21 met conventional psychometric properties.

Using CFA, we fit the data using three different models: a unidimensional model, second-order model, and first-order model. The unidimensional model, with all 23 items loading on a single latent variable (see Fig. 1) did not show a satisfactory model fit. The second-order model, with all 23 items loaded on the three respective latent variables (i.e. clutter, difficulty discarding, acquisition) proposed by Frost et al. [12] (see Fig. 2), was empirically under-identified. This could be due to (1) high cross-loading of items on more than one factor, or (2) data from the current sample being too ‘far away’ from the specified model, resulting in under-identification despite positive degree of freedom [43]. In the first-order model, items loaded on three respective latent variables (*Clutter*, *Difficulty discarding*, *Acquisition*), which were correlated with each other (see Fig. 3). The model yielded mediocre model fit in the final solution. Two items with low factor loadings, item 2 (‘control over urges to acquire’) and item 4 (‘control over urges to save’), were removed in the final solution of the CFA, resulting in the modified SIR-21.

These two items, item 2 (‘control over urges to acquire’) and item 4 (‘control over urges to save’) were also removed in previous studies conducted in Chinese samples [22, 23]. Thus, the present results are not surprising given our majority Chinese sample. The inability of these items to demonstrate strong factor loadings in the Chinese college samples [22, 23], and current majority Chinese sample suggest that modification of these items may be required for use in Chinese samples. However, given that these two items are reverse-coded, we are unable to conclude if these findings are due to participants’ inability to grasp the wording of the items, or due to their difficulty comprehending items related to ‘self-control’. It is possible that the role of self-control is differentially emphasized in collectivistic cultures [44]. More specifically, perceived sense of self-control among Asians may be more likely to be characterized by the perceived ability to change one’s thoughts and actions in order to conform to the environment, rather than changing the environment to fit one’s own goals or pursuits [44, 45]; the latter is more likely associated with Western populations. Hence, self-control (turning inward versus outward to actively change the environment) might influence hoarding behavior differently in Asian populations. In other words, these two ‘self-control’ items might be capturing a different construct in the current samples compared to the U.S. sample in Frost et al.’s [12] study. Further research with Chinese or similar samples, including studies that use qualitative methodology, may illuminate the role of self-control in hoarding in these cultures.

In addition, in Tang et al.’s [23] study, failure to obtain a good model fit from CFA led them to adopt a data-driven approach (i.e., exploratory factor analysis) to examine the underlying factor structure in their Chinese sample. They extracted two factors – *Difficulty Discarding – Acquisition*, and *Clutter* (Tang et al., 2012). Tang et al. [23] stated that cultural elements might have affected understanding of the construct of difficulty discarding, as Chinese individuals tend to perceive both ‘not discarding’ and ‘acquiring new things’ as ‘possessing’. Although acquiring new things and not discarding old things represent active and passive acts in hoarding respectively, Chinese tend to integrate these two concepts, as described by the adage, ‘those we do not waste are those we gain’ [23]. This discrepant view of saving/acquiring behavior in Chinese culture may explain the mediocre model fit obtained in the current study. However, we were unable to verify this hypothesis using both CFA and exploratory factor analysis by splitting our sample (*N* = 500), as the split-half sample size was not sufficient for stable convergence of a solution.

Moreover, the perception toward discarding possessions may be different across cultures. As discussed in Du and Jing’s work [46], there is a strong emphasis on saving for future use in Chinese culture. Indeed, practicing frugality and saving possessions and other commodities for future use are seen as virtues among Chinese individuals [46]. Hence, it is possible that in Chinese culture, discarding is considered a wasteful habit rather than a “difficult” behavior rooted in attachment to possessions. As such, items may need to be revised to reflect discarding in a way that is both culturally and phenomenologically consistent. For example, instead of asking about difficulty discarding, questionnaire items could instead be framed in terms of actual frequency of behavior (e.g., "How often do you discard items you are unlikely to use in the future?"). In addition, individuals with hoarding problems in this culture may also perceive saving behaviors positively. Due to the heterogeneous cultural backgrounds of the current sample, future studies should be done to examine differences across the various ethnic groups (i.e. Malays, Indians).

The housing structure of Singapore makes it a unique setting for the presentation of hoarding. Similar to the wider Singapore resident population [25], more than 80 % of our current psychiatric sample reported staying in HDB flats (87.7 %). Sixty-nine percent of them were staying with their immediate family (see Table 1.). The lack of strong associations between the CIR and the ADL-H and SI-R, as compared to the SCI could be in part attributed to the housing structure in Singapore, since the close proximity of family members staying in the same household may deter or diminish clutter accumulated by individuals with hoarding problems.

Limitations of current study should be noted. Participants did not receive a formal DSM-5 diagnosis of hoarding disorder, and caution must be exercised in generalizing our findings to clinical populations. However, using the recommended cutoff by Frost, Steketee, and Tolin (unpublished data, cited in [14]), we found that 30.9 % of the current sample met criteria for clinically significant hoarding, and 12.9 % of the current sample exhibited significant levels of difficulty discarding, clutter and excessive acquisition (see [47]). In addition, the current sample only included individuals who were proficient in English, and may not be fully representative of the Singaporean psychiatric outpatient population.

Future research may benefit from focusing on the development of the SI-R and other hoarding scales for use in cultures outside the U.S. and in languages other than English. Qualitative studies that reveal underlying hoarding constructs unique to diverse populations can also be helpful in establishing and improving the external validity of the SI-R. Finally, comparison and evaluation of construct validity across diagnoses as well as between non-clinical and clinical hoarding samples using larger sample sizes is suggested.

## Conclusion

A CFA of the SI-R using a Singaporean psychiatric sample based on Frost et al.’s [12] original three-factor model yielded a mediocre model fit, suggesting that the phenomenology of hoarding in the current sample might not have been sufficiently captured by the SI-R. Cultural differences may play a significant role in the perception of saving, acquiring, and discarding behavior. Despite inconclusive results on its factor structure, the SIR-21 and subscales showed convergent and discriminant validity given its correlations with hoarding-related constructs, (SCI, CIR, ADL-H), anxiety symptoms (BAI), and depressive symptoms (BDI-II). Future studies should examine the qualitative aspect of the presentation of hoarding in diverse cultures, as well as further evaluate the psychometric properties of the SI-R in non-Western contexts.

## Abbreviations

ADL-H: Activities of daily living scale for hoarding
BAI: Beck anxiety inventory
BDI-II: Beck depression inventory
CFA: Confirmatory factor analysis
CFI: Comparative fit index
CIR: Clutter image rating
df: Degree(s) of freedom
DSM-5: Diagnostic and statistical manual of mental disorders, Fifth edition
DSM-IV: Diagnostic and statistical manual of mental disorders, Fourth edition
HDB: Housing Development Board
HRS: Hoarding Rating Scale
IMH: Institute of Mental Health
NNFI: Non-normed fit index
RMSEA: Root mean square error of approximation
SCI: Saving cognitions inventory
SI-R: Saving inventory – revised
SRMR: Standardized root mean square residual
TLI: Tucker-Lewis Index
